# Retraction: Fisheries and Marine Animal Populations: Learning from the Long Term

**DOI:** 10.1371/annotation/6df4032b-49ed-4e74-a960-37a107827dd5

**Published:** 2011-02-03

**Authors:** David J. Starkey, Tim D. Smith, Michaela Barnard

It has been brought to the attention of the PLoS ONE Editors that a substantial part of the text in this article was appropriated from text in the following publication:

Holm P, Marboe AH, Poulsen B, MacKenzie BR (2010) Marine animal populations: A new look back in time. In: McIntyre AD, editor. Life in the world’s oceans. London (WileyBlackwell), 2010.

The first author of the article, Dr Starkey, has claimed responsibility for the problem that has led to the withdrawal of this overview.

PLoS ONE therefore retracts this article due to the identified case of plagiarism.

